# Suboptimal control for patients with type 2 diabetes in the Central Chronic Medicine Dispensing programme in South Africa

**DOI:** 10.4102/phcfm.v13i1.2648

**Published:** 2021-03-24

**Authors:** Patrick Ngassa Piotie, Elizabeth M. Webb, Paul Rheeder

**Affiliations:** 1 School of Health Systems and Public Health, Faculty of Health Sciences, University of Pretoria, Pretoria, South Africa; 2 Department of Internal Medicine, Faculty of Health Sciences, University of Pretoria, Pretoria, South Africa

**Keywords:** audit, CCMDD programme, glucose control, primary health care, SEMDSA guidelines, type 2 diabetes

## Abstract

**Background:**

In South Africa, the Central Chronic Medicine Dispensing and Distribution (CCMDD) programme allows stable patients with non-communicable diseases, including type 2 diabetes mellitus (T2DM), to collect their medication from a pick-up location near their home, thus avoiding long waiting times and travel expenses. The CCMDD programme aims at improving patient retention and adherence through better access to medicines, resulting in better health outcomes.

**Aim:**

We assessed whether patients with T2DM enrolled in CCMDD achieved the recommended targets for glycaemic, blood pressure (BP) and lipid control.

**Setting:**

City of Tshwane, South Africa.

**Methods:**

We reviewed the records of 198 T2DM patients enrolled in CCMDD and assessed their control of haemoglobin A1c (HbA1c), BP and lipids.

**Results:**

Most of the records reviewed belonged to women (64.7%), African (89.9%), hypertensive (82.7%) and to patients exclusively on oral antidiabetic agents (98.5%). Patients were, on average, 57.7 (s.d. = 12.1) years old and had participated in the CCMDD programme for, on average, 2 years. The mean HbA1c was 8% (s.d. = 2). Glycaemic control was achieved by only 29.2% of patients, and 49% of patients had HbA1c between 7% and 9%. Ninety-three patients (66%) had achieved the total cholesterol target, 57.4% achieved BP targets and 6.9% had achieved the low-density lipoprotein cholesterol target.

**Conclusion:**

A small group of patients achieved the targets for glycaemic, BP and lipid control. Despite improved accessibility to medication, the CCMDD is not synonymous of improved clinical outcomes. Future research should ascertain the factors associated with suboptimal control for these patients.

## Introduction

In Africa, an increasing number of people with type 2 diabetes mellitus (T2DM) has been reported day by day. This increase has been linked to various factors, including lifestyle changes, urbanisation and the growing consumption of processed foods coupled with an increasing prevalence of obesity.^1,2^ Currently, an estimated number of 19.4 million African adults, aged 20–79 years, are living with mostly type 2 diabetes. The International Diabetes Federation (IDF) estimates that this number will increase to 47.1 million (142.9% increase) by 2045.^3^ Similar trends are being observed in South Africa. The 2019 IDF report estimates that 4.6 (1.4–5.3) million adults have diabetes in South Africa, with a national prevalence of 12.8%.^3^ Of these 4.6 million adults, an estimated number of 2.4 million adults (52.2%) are undiagnosed. In 2019, South Africa had reported 89 834 diabetes-related deaths, the highest in Africa.^3^ South African statistics reported that diabetes was second to tuberculosis as the most common natural cause of death.^4^

Several activities across Africa aim to improve the management of type 2 diabetes and related comorbidities.^1^ In South Africa, various programmes support the management, monitoring and adherence to prescribed medicines of people with type 2 diabetes.^1,5,6^ One such initiative is the Central Chronic Medicine Dispensing and Distribution (CCMDD) programme, a decentralised chronic medication delivery system enabling public sector patients to collect chronic medication closer to their homes rather than having to travel to hospitals or primary healthcare clinics.^1,6,7^ The South African National Department of Health initiated the programme in February 2014 to ease the burden of primary healthcare facilities and staff, as well as to reduce long waiting times and poor stock management that led to medicine shortages and poor service delivery.^7,8^ Initially, the CCMDD programme aimed to enhance access to antiretroviral drugs, and it was subsequently expanded to include patients with chronic conditions, such as type 2 diabetes and hypertension.^6^

Patients are eligible for the CCMDD programme if they are older than 18 years of age, able to provide consent and are stable on chronic medication. Being stable on medication is defined as being on the same treatment regimen for at least 12 months, with the two most recent laboratory results being normal. Eligible patients cannot be under tuberculosis medication or any other medical condition requiring regular clinical consultations.^9^ Once enrolled, patients can routinely collect pre-packaged chronic medication from registered pick-up points, such as shops, places of worship, community halls or schools.^8,10^ Patients in the CCMDD programme are required to go for clinical assessments at a healthcare facility every 6 months.

According to various reviews, the CCMDD programme improves facility congestion, reduces patient travel and long waiting times, and improves treatment adherence and patient retention.^1,6,10,11^ One of the studies concluded that the CCMDD programme reduces medicine stock-outs and improves patient outcomes.^8^ Health authorities have also claimed the success of the CCMDD programme in some South African provinces.^12^ Notwithstanding these promising results and claims, we could not find any study focussing on the clinical outcomes of patients with type 2 diabetes in the CCMDD programme.

International trials demonstrate that glycaemic control is important for preventing both acute and long-term complications of diabetes mellitus.^13,14^ Despite clear professional guidelines such as those provided by the Society for Endocrinology, Metabolism and Diabetes South Africa (SEMDSA) 2017,^15^ glycaemic control is often suboptimal or poor in South Africa.^16,17,18,19,20^ This is concerning because people with poorly controlled diabetes may require greater medical intervention if they acquire viral infections, including the novel coronavirus disease 2019 (COVID-19), and poorly controlled type 2 diabetes is associated with a higher death rate amongst COVID-19 patients.^21^

In this article, we describe the clinical outcomes of patients with type 2 diabetes who are enrolled in the CCMDD programme in the City of Tshwane. We assess whether these patients with type 2 diabetes achieve the 2017 SEMDSA recommended targets for glycaemic, blood pressure (BP) and lipid control. Policymakers could use the results of this study as a basis for a full evaluation in order to determine whether the patients with type 2 diabetes enrolled are truly benefitting from the CCMDD programme.

## Research methods and design

### Study design

This research study stemmed from a clinical audit conducted between February and May 2019. The audit identified gaps in diabetes management and care, as well as missed opportunities for therapy intensification. This study forms part of the Tshwane Insulin Project (TIP), a 5-year translational research programme at the University of Pretoria. The TIP aimed to improve diabetes management at primary healthcare in South Africa.

### Setting

The study population included patients from 23 primary healthcare facilities, including 20 clinics and three community healthcare centres in the Tshwane Health District. The Tshwane Health District is situated in the northern part of Gauteng Province in South Africa and has the same geographical boundaries as that of the City of Tshwane Metropolitan Municipality.

Although diabetes mellitus is included in the Integrated Chronic Disease Management (ICDM) model, its prevalence in the Tshwane Health District is unknown.^22^ A stepwise approach for managing type 2 diabetes is outlined in the ‘Standard Treatment Guidelines and Essential Medicines List of South Africa’.^23^ The guidelines focus on nurse-initiated treatment and provide algorithms for both doctors and professional nurses. Most people with type 2 diabetes should consult a healthcare professional at least four times per year. In practice, most patients with diabetes attend primary healthcare facilities every month for testing of random blood glucose and BP levels, weight checks and to collect their medications.^22^ Patients who are eligible for the CCMDD programme may collect their medication every month from a decentralised location or from the clinic pharmacy and consult with a healthcare professional every 6 months in order to assess disease control and review medication.

### Study population

For this audit, we selected medical records of patients with type 2 diabetes who received care at a primary healthcare facility and who were enrolled in the CCMDD programme. Patients with type 2 diabetes are eligible to enrol in the CCMDD programme if they have two consecutive levels for fasting plasma glucose (FPG) normal and two consecutive levels for BP normal (if hypertensive). Trained fieldworkers visited the selected healthcare facilities. Using a consecutive sampling technique, they selected the first 10–15 medical records per facility of adults with type 2 diabetes who were already on CCMDD.

### Data collection

We used Qualtrics (Provo, UT) to design a data extraction sheet to collect data from patient medical records. Trained fieldworkers were equipped with electronic tablets to collect and record data, which included demographics, clinical measurements (BP) and laboratory measurements, such as haemoglobin A1c (HbA1c) and lipids (total cholesterol and low-density lipoprotein (LDL)). We could not calculate the body mass index because most patient records lacked weight and height measurements.

Data collection was limited by the poor quality of medical records, and facilities not having diabetes registries hampering our ability to identify the medical records of patients with type 2 diabetes. Not all medical records had laboratory results, further limiting the collection of data.

### Data analysis

Data were analysed using STATA version 15.1 (Statacorp LP, College Station, TX). Patient characteristics were summarised by descriptive statistics. Categorical variables are reported with frequencies and percentages. Continuous variables are reported with means and standard deviations or medians and interquartile ranges. The proportion of patients who met the treatment goals are reported with 95% confidence intervals.

In this study, we used the targets set out by the 2017 SEMDSA guidelines for the management of T2DM as the reference standard.^15^ Haemoglobin A1c used was ≤ 7%.^15^ The BP target was set as < 140/90 mmHg. Targets for cholesterol were as follows: total cholesterol < 4.5 mmol/l; LDL cholesterol < 1.8 mmol/l.^15^

### Ethical considerations

This study was approved by the University of Pretoria’s Faculty of Health Sciences Research Ethics Committee (Ethics Reference: 496/2018) and the Tshwane Research Committee (NHRD Number: GP_201810_049). Access to medical records was granted by the custodians of the data, namely, the health district authorities and the health facility managers.

## Results

We audited 232 patient medical records from 23 primary healthcare facilities. We excluded 34 records because of missing data and retained the records of 198 patients who had enrolled in the CCMDD programme between 2014 and 2019. The patient characteristics are displayed in Table 1. The mean age of the patients was 57.7 (s.d. = 12.1) years, with a median duration of diabetes of 5.0 years (CI: 4% – 6%). Most of the patients were women (64.7%), African (89.9%) and exclusively on oral antidiabetic agents (98.5%). At the time of the study, participants had been enrolled in the CCMDD programme for 2.0 years, on average, and 82.7% had hypertension. The most recent prescription in medical records indicated that 150 (75.8%) patients were receiving statins for dyslipidaemia.

**TABLE 1:** Demographics and clinical characteristics of a sample of patients with type 2 diabetes (*N* = 198) in the CCMDD programme in Tshwane Health District, South Africa.

Patient characteristics	*n*	%
**Gender**		
Women	128	64.7
Men	70	35.3
**Age, years**		
Mean (s.d.)	57.7	12.1
18–50	56	28.5
51–65	91	46.2
> 65	50	25.4
**Ethnicity**		
African	178	89.9
Other	17	10.1
**Duration of diabetes, years**		
Median (IQR)	5.0	3.0 – 7.0
< 5	35	17.7
5–10+	49	24.7
Not recorded	114	57.6
**Diabetes medication**		
Oral agents only	195	98.5
Oral and insulin	3	1.5
**Time on CCMDD programme, years**		
Median (IQR)	2.0	1.0 – 3.0
< 1–2	123	73.7
3–5	44	26.3
Hypertension	163	82.7
Dyslipidaemia	150	75.8

Other, Asian/Indian, Mixed race and white people; s.d., standard deviation; IQR, interquartile range.

Of 198 patients, 144 (72.7%) patients recorded HbA1c measurements for the previous year with a mean HbA1C of 8.0% (s.d. = 2.0) (Table 2). Blood pressure was recorded for 99.5% of patients at their most recent clinic visit, however, only 14.6% of patients had an LDL cholesterol test performed in the previous year.

**TABLE 2:** Diabetes control parameters in a population of patients enrolled in the CCMDD programme in Tshwane.

Diabetes parameters	Tests done (*N* = 198)		Mean value	s.d.	Range (Min. – Max.)
	*n*	%			
HbA1c (%)	144	72.7	8.0	2.0	4.2 – 18.9
**Lipids (mmol/L)**					
LDL cholesterol	29	14.6	2.9	1.0	1.5 – 5.4
Total cholesterol	141	71.2	4.2	1.0	1.9 – 7.2
**Blood pressure (mmHg)**					
Systolic BP	197	99.5	134.1	16.6	100 – 191
Diastolic BP	197	99.5	79.3	10.9	40 – 105

CCMDD, Central Chronic Medicine Dispensing; BP, blood pressure; LDL, low-density lipoprotein; s.d., standard deviation.

**FIGURE 1:**
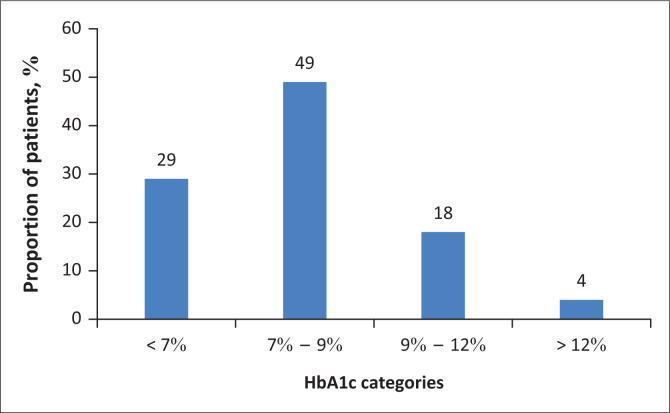
Distribution of haemoglobin A1c values in a population of Central Chronic Medicine Dispensing patients with type 2 diabetes in Tshwane Health District, South Africa.

Of the patients who had HbA1c measurements, only 29.2% (CI: 21.9% – 37.3%) met the 2017 SEMDSA target of HbA1c < 7% (Table 3). Almost half (49%) of the patients reported HbA1c values between 7% and 9% (Figure 1). Only 57.4% (CI: 50.1% – 64.4%) of the patients achieved the BP target (< 140/90 mmHg); 67.0% (CI: 60.0% – 73.5%) and 84.8% (CI: 79.0% – 89.5%) met the targets for systolic and diastolic BP, respectively. More than half of the patients met their total cholesterol target (66%, CI: 57.5% – 73.7%), but only two (6.9%, CI: 0.9% – 22.8%) of the 29 patients with LDL cholesterol levels met the LDL target.

**TABLE 3:** Proportions of patients enrolled in the CCMDD programme in Tshwane Health District, South Africa meeting the SEMDSA 2017 targets for diabetes control.

Diabetes parameters	2017 SEMDSA	Target attained		95% CI (%)
		*n*	%	
HbA1c	< 7	42	29.2	21.9 – 37.3
**Lipids (mmol/L)**				
LDL cholesterol	< 1.8	2	6.9	0.9 – 22.8
Total cholesterol	< 4.5	93	66.0	57.5 – 73.7
**Blood pressure (mmHg)**				
Combined BP	< 140/90	113	57.4	50.1 – 64.4
Systolic BP	< 140	132	67.0	60.0 – 73.5
Diastolic BP	< 90	167	84.8	79.0 – 89.5

SEMDSA, Society for Endocrinology, Metabolism and Diabetes South Africa; CI, confidence interval; BP, blood pressure.

## Discussion

In this study, we assessed whether South African primary care patients with type 2 diabetes enrolled in the CCMDD programme were achieving the recommended 2017 SEMDSA targets for diabetes management. These include targets for glycaemic (HbA1c), BP and lipid control. From the patient records, we observed that as a whole, patients enrolled in the CCMDD programme reported suboptimal diabetes control, with only 29.2% of patients achieving glycaemic targets, 57.4% of patients achieving BP targets and 6.9% of patients achieving LDL cholesterol targets. Similar to other studies focussing on patients with type 2 diabetes in the South African primary healthcare sector, the patients in this study were predominantly African and women, with a reported mean age of 53.0–59.4 years.^16,17,18,24^

Historically, glycaemic control has been suboptimal in South Africa’s primary healthcare sector.^25,26^ In this study, the proportion of patients who reported HbA1c values as a measure of glycaemic control were quite high (72.7%) compared with those in other South African studies^24,27^ and were similar to glycaemic testing rates reported in a dozen of primary healthcare facilities in the Tshwane district.^16^ The results of this study confirmed poor glycaemic control amongst patients with type 2 diabetes in South Africa, with only 29.2% of patients achieving control.^16,17,18,24^ This is concerning because to be eligible for enrolment in the CCMDD programme, these patients should have been stable controlled patients. The fact that the largest proportion of patients had not achieved glycaemic control indicates that access to medicines is not the only factor influencing glycaemic control in this population.

For people living with diabetes, glycaemic control is improved by various factors, including higher socio-economic status, better dietary knowledge, and higher self-efficacy and empowerment.^28^ Self-efficacy is defined as the patient’s personal judgement of his or her confidence in performing activities related to diabetes self-management, for example, maintaining a regular exercise routine, accessing medical services or testing blood glucose levels.^29^ In South Africa, primary healthcare patients frequently have insufficient knowledge regarding diabetes, and self-care as well as insufficient means to comply with the demands of the disease, resulting in almost one out of two patients failing to practise any form of lifestyle modification.^30^ Primary care patients experience multiple barriers to effective self-management and behaviour change, including poor health literacy and lack of self-efficacy.^31^ These barriers may explain why there were a small proportion of patients with glycaemic control in this study. One of the ways to improve health literacy and self-efficacy is to having more frequent contact with healthcare providers. Murphy et al.^31^ found that patients from the public primary healthcare sector desire for greater assistance and support from their healthcare providers. Unfortunately, patients who are enrolled in the CCMDD programme had less contact with healthcare providers, which may have contributed to the lower-than-expected rate of control.

Patients enrolled in the CCMDD programme require management that recognises the importance of managing hypertension, as well as other comorbidities related to diabetes. In addition to glycaemic control, the management of type 2 diabetes should focus on lowering levels of LDL cholesterol and BP to prevent cardiovascular diseases.^14,32^ In this study, 82.7% of patients were reportedly hypertensive. Other South African studies have also reported a high prevalence of hypertension in people with type 2 diabetes (79% – 89%).^16,17,18,33^ In contrast to other studies that reported poorer outcomes, more than half of the patients in this study (57.4%) achieved their BP targets.^17,18,34^ In a review of 14 studies from 19 different countries, BP control is often the least-achieved target.^32^ This may be because treating diabetes-associated risk factors, including hypertension, is inadvertently under-emphasised.^32^ In South Africa, suboptimal BP control may be attributed to inadequate treatment,^18^ clinical inertia,^19^ poor compliance with diabetes management guidelines,^24^ as well as patient factors, such as lack of knowledge and low self-efficacy.^10,30,31^

Dyslipidaemia was prevalent in the type 2 diabetes patients (75.8%) in this study. We analysed total cholesterol levels for 71.2% of patients in this study, which was similar to testing rates in other South African studies.^24^ For patients with diabetes, clinical trials have clearly demonstrated that lowering LDL cholesterol levels, particularly with statin treatment, reduces the risk of major cardiovascular events.^35^ The 2017 SEMDSA guidelines state that LDL is the primary target of lipid-lowering therapy.^15^ Despite these guidelines, only 29 patients (14.6%) in this study had reported a recent LDL test result. Webb et al.^16^ reported a testing rate of 53.9%, whilst Pinchevsky et al.^18^ reported 58.5% of LDL tests performed in primary care facilities. In this study, two patients (6.9%) met the LDL target compared with 93 (66.0%) who met the total cholesterol target. Pinchevsky et al.^18^ and Webb et al.^16^ reported more patients (56.3% and 16.0%, respectively) reaching their LDL targets. Our audit of patients enrolled in the CCMDD programme revealed an inconsistent LDL testing protocol, which needs to be addressed by healthcare managers. Poor testing protocols suggest that healthcare professionals are not adhering to diabetes management guidelines, putting patients at risk of developing long-term complications.^24,27^

### Recommendations for the Central Chronic Medicine Dispensing programme for people with type 2 diabetes

The CCMDD programme was designed to improve access to chronic medication for stable patients. The benefits to patients include reduced clinical visits, waiting times and travel expenses. The benefits to the healthcare system include facility decongestion and reduction of medicine stock-outs.^6,8,11^ We found that patients with type 2 diabetes enrolled in the CCMDD programme did not have better glycaemic, BP and lipid control when compared with primary healthcare patients from other South African studies. Although we did not investigate glycaemic control prior to enrolment, it is likely that some patients enrolled in the CCMDD programme were not stable to begin with. The question that arises is whether people with type 2 diabetes truly benefit from the CCMDD programme, especially when measuring clinical outcomes.

Currently, the CCMDD programme evaluates the glycaemic control of potential patients using two consecutive FPG measurements.^9^ Fasting plasma glucose measures an individual’s ability to regulate blood glucose levels in the absence of the dietary glucose input.^36^ Fasting plasma glucose is not a reliable indicator of glycaemic control because it measures blood glucose levels at a single point in time.^37^ Measuring HbA1c is considered the gold standard for assessing glycaemic control, and studies have shown that HbA1c is difficult to predict from FPG values.^38,39^ HbA1c provides an indication of blood glucose concentrations over the previous 2–3 months.^36^ To assess eligibility, we recommend that the CCMDD programme evaluates glycaemic control of people with type 2 diabetes using two consecutive target HbA1c values (< 7%) instead of FPG.

Once enrolled in the CCMDD programme, patients with type 2 diabetes have less contact with the public healthcare system than those frequently attending clinics. Patients in the CCMDD programme have to perform a number of self-management activities and make daily decisions contributing to their well-being whilst dealing with the demands and burdens of diabetes, and thus, require high levels of self-efficacy. Not assessing self-efficacy before being enrolled in the CCMDD programme may disadvantage patients because low self-efficacy will lead to treatment failure and poor clinical outcomes.^40^ Self-efficacy has been strongly associated with healthy eating and adequate physical activity.^41^ We recommend that the self-efficacy of potential patients should be evaluated before enrolling in the CCMDD programme in order to ensure that patients are able to perform the required self-management activities.

The successful management of chronic diseases depends on effective, systematic and interactive communication between patients and healthcare professionals.^42^ People living with diabetes need to be taught the skills and informed about how to best manage the disease on a day-to-day basis.^15,43^ Diabetes self-management education and support (DSMES) is the ongoing process of facilitating the knowledge, skills and ability necessary for diabetes self-care, as well as activities that assist a person in applying and sustaining the behaviours needed to manage his or her condition on an ongoing basis.^44^ Critical time points and structured care for providing DSMES have been well documented.^44,45^ These include (1) at diagnosis, (2) annually, (3) when complicating factors occur and (4) during transitions in care. In the present context, we recommend that DSMES should be provided when patients are enrolled in the CCMDD programme. Furthermore, DSMES should not be seen as a once-off event but rather a lifelong necessity, and therefore, healthcare managers should explore strategies to incorporate DSMES into the CCMDD programme.^15^ Already, innovative education programmes for patients with hypertension on the CCMDD programme have been tested.^46^

### Limitations of the study

This research study was the first audit of diabetes control in patients enrolled in the CCMDD programme. The cross-sectional design only reflects the once-off measurements recorded at the time of the study. A prospective study of patients with diabetes in the CCMDD programme, in the form of a large-scale evaluation, would provide more information in terms of disease status at enrolment into the programme, as well as factors that influence control over time. A future evaluation of the programme could make use of control groups or could be a cohort study.

This study looked at a relatively small number of patients with type 2 diabetes enrolled in the CCMDD programme, predominantly women and African in an urban setting. The generalisability of the findings may not be entirely applicable to all patients with diabetes in the programme across South Africa. A strength of the study, however, was the spread of the cohort of patients over 23 primary healthcare facilities.

The quality of patient medical records was not always satisfactory, and therefore, this study may have missed important information because of missing data.

## Conclusion

A small proportion of patients enrolled in the CCMDD programme achieved the recommended targets for glycaemic, BP and lipid control. The findings of this study suggest that the ability to access medicines remotely does not guarantee good health outcomes compared with other primary healthcare populations. We recommend that the CCMDD programme for people with diabetes should consider revising how patients with diabetes are selected, assessing self-efficacy before enrolment, and should include additional accompanying measures for patient empowerment and education.
